# Bringing to light unnoticed data on the genetic and host diversity of ungulate *Plasmodium*

**DOI:** 10.1016/j.ijppaw.2025.101104

**Published:** 2025-06-18

**Authors:** Boris Kevin Makanga, Larson Boundenga, Paul Yannick Bitome-Essono, Céline Arnathau, Virginie Rougeron, Franck Prugnolle

**Affiliations:** a Institut de Recherches en Ecologie Tropicale, Libreville, Gabon; b Centre International de Recherches Médicales de Franceville, Franceville, Gabon; c Laboratoire MIVEGEC, Université de Montpellier, CNRS/IRD/UM, IRD Montpellier, Montpellier, France; d International Research Laboratory, REHABS, CNRS-NMU-UCBL, George Campus, Nelson Mandela University, Madiba drive, 6529, George, South Africa; e Sustainability Research Unit, George Campus, Nelson Mandela University, George Campus, Madiba drive, 6529, George, South Africa

## Abstract

The genus *Plasmodium*, best known for causing malaria in humans, also infects a diverse array of vertebrates, including ungulates. Despite the first report of *Plasmodium* in ungulates dating back to 1913, research on these parasites has remained scarce, largely overshadowed by studies on primate, avian, and rodent *Plasmodium*. A century later, in 2016, three independent publications renewed interest by reporting the first genetic sequences of ungulate *Plasmodium* from different host species and continents. Since then, several studies have explored their genetic diversity across various host species and geographic regions. Among these, two studies investigated *Plasmodium* in African forest and savanna ungulates, identifying several new host species, particularly within the genera *Tragelaphus*, *Syncerus*, and *Cephalophus*. However, these findings remained largely unnoticed by the *Plasmodium* research community, as the primary focus of the publications was on xenosurveillance (the use of hematophagous flies and their blood meals to detect pathogens) rather than malaria parasites *per se*. Here, we reanalyze these overlooked data to clarify the evolutionary relationships of ungulate *Plasmodium*. Phylogenetic analyses confirm that these parasites form a monophyletic group, distinct from *Plasmodium* infecting primates, rodents, and bats. Within this group, two main clades were identified, encompassing parasites from various ungulate hosts. While *Cephalophus* parasites cluster with previously described sequences, those from *Tragelaphus* and *Syncerus* form distinct lineages, likely representing novel species. Expanding knowledge of ungulate *Plasmodium*, particularly in under-explored regions and host species, is crucial for understanding the evolution and diversity of these parasites.

## Highlights

• Bringing to light “unnoticed” sequences on ungulate *Plasmodium.*
• Ungulate *Plasmodium* form a monophyletic group, distinct from other mammal *Plasmodium.*
• Ungulate *Plasmodium* are subdivided into two subclades.
• Phylogenetic analyses reveal novel *Plasmodium* lineages in *Tragelaphus* and *Syncerus.*

## Graphical abstract

**Figure:**
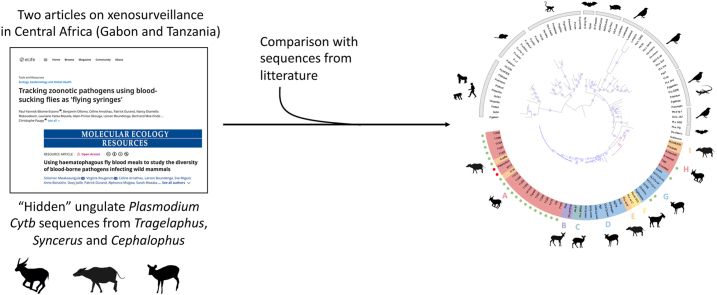

## 1 Introduction

The genus *Plasmodium*, best known for causing malaria in humans, also infects a wide range of vertebrates, including reptiles, birds and mammals (Perkins and Schaer, 2016). Among mammals, *Plasmodium* species have been described to infect primates, rodents, bats, and ungulates (Perkins and Schaer, 2016). While the first publication on ungulate *Plasmodium* dates back to the beginning of the 20th century, the diversity and host range of *Plasmodium* species infecting ungulates has remained largely unexplored, likely overshadowed by research on *Plasmodium* of primates, birds and rodents.

The first description of a malaria parasite (*Plasmodium cephalophi*) in an ungulate, an African duiker (*Sylvicapra grimmia* previously named *Cephalophus grimmi*) from Malawi, dates back to 1913 (Bruce et al., 1913), but this was not until 1966 that this parasite was rediscovered (Keymer, 1966). Around the same period (1919), another ungulate *Plasmodium* species, *Plasmodium bubalis*, was described in Asian water buffaloes (Sheather, 1919). Following this, additional haemosporidian species of ungulates have been microscopically described, but these studies remained limited in terms of the number of ungulate species screened, the number of individuals analysed per host species and the geographic areas covered by these studies. *Plasmodium caprae* was thus described from some African goats (*Capra hircus*) (De Mello, 1923); *Plasmodium limnotragi* from a marshbuck (*Tragelaphus spekii* previously named *Limnotragi spekei*) in Africa (van den Berghe, 1937); *Hepatocystis fieldi* from the hippopotamus (*Hippopotamus amphibious*) (Garnham, 1958) and *Plasmodium traguli* from the Mouse deer in Asia (Garnham and Edeson, 1962). The most recent one, in the early 1980's, was *Plasmodium odocoilei* from a white-tailed deer (*Odocoileus virginianus*) in North America (Garnham and Kuttler, 1980).

Then, all studies on ungulate *Plasmodium* ceased for more than 30 years, until 2016, when three studies reinvestigated the diversity of ungulate *Plasmodium* across different regions and host groups using molecular tools (Boundenga et al., 2016; Martinsen et al., 2016; Templeton et al., 2016a). Although these studies likely re-discovered previously described *Plasmodium* species (e.g. *P. odocoilei* in white-tailed deers, *P. cephalophi/P. brucei* in duikers (*Cephalophus* species) and *P. bubalis* in water buffaloes), they also extended the diversity of known hosts for each *Plasmodium* species and, more importantly, provided insights into their genetic diversity and phylogenetic relationships, both among themselves and with other extant malaria parasites. These studies also reignited the interest for ungulate *Plasmodium* as several studies were published in the following years, especially from South American and Asian ungulates (Asada et al., 2018; Dos Santos et al., 2018; Kaewthamasorn et al., 2018; Nguyen et al., 2023; Ulloa et al., 2024). The emerging picture from all these studies is that malaria parasites infecting ungulates cluster within a monophyletic clade, genetically distinct from other mammalian *Plasmodium* (Perkins and Schaer, 2016; Templeton et al., 2016b). This clade is indeed more genetically related to bird or lizard *Plasmodium*, as well as to the specific bat genus *Polychromophilus*, than to all the other known mammalian *Plasmodium* or *Hepatocystis* parasites infecting primates, rodents and bats (Boundenga et al., 2016; Poofery et al., 2023; Templeton et al., 2016b). Within this ungulate clade, the lineages formed by the parasites from African duikers, Asian water buffaloes, African goats and American cervidae seem intermixed, suggesting multiple independent host switching events in these host species, rather than co-divergence with their hosts (Perkins and Schaer, 2016; Templeton et al., 2016b). The genetic diversity of *Plasmodium* parasites infecting ungulates uncovered in these studies surpasses the number of species previously identified based solely on morphology (Perkins and Schaer, 2016; Templeton et al., 2016b), thus suggesting the existence of a larger than previously recognized diversity of species in ungulate *Plasmodium*.

Shortly after 2016, another study published results on the diversity of *Plasmodium* from a large diversity of forest ungulates from Gabon (Bitome-Essono et al., 2017). The focus of the study was not the ungulate *Plasmodia per se* but the description of a new methodology: the use of hematophagous flies as “flying syringes” to obtain non-invasively blood samples of vertebrate hosts to study their blood-borne pathogens. In this publication, the authors chose to screen extant malaria parasites from the collected blood samples obtained from the blood-engorged flies because of their presence in a large range of vertebrate hosts in rather high prevalence. This approach enabled the first molecular detection and sequencing of *Plasmodium* infections in ungulates from previously unstudied genera, such as those of the genus *Tragelaphus* (e.g. *Tragelaphus spekii*, the marshbuck) and *Syncerus* (*Syncerus caffer*, the African buffalo). A similar xeno-surveillance approach was applied in a savannah ecosystem of Tanzania a couple of years later (Mwakasungula et al., 2022). In this study, using the same non-invasive approach, the authors analysed the diversity of pathogens (viruses, bacteria and protozoa, including extant malaria parasites) from a large diversity of hosts, of which several savannah ungulate species. Obtained results showed that one of these ungulate species, the greater kudu, *Tragelaphus strepsiceros*, harboured *Plasmodium* infections.

Unfortunately, despite the importance of these results for the understanding of the evolution of ungulate *Plasmodium*, until now, all these findings have remained unnoticed by the community of researchers working on *Plasmodium* parasites as none of the studies on ungulate *Plasmodium* published after these two publications cited them or included the sequences in their phylogenetic analyses. This is likely due to the fact that the neglected sequences were reported in studies on xenosurveillance, rather than in malaria-focused publications. As a result, a significant portion of the known genetic diversity of ungulate *Plasmodium* has remained hidden so far.

The present article aims to bring to light this unnoticed diversity of ungulate *Plasmodium* and integrate it with the rest of the genetic diversity that has been discovered so far in ungulates. By compiling and analysing these “unnoticed” data, this study seeks to refine the phylogenetic placement of ungulate *Plasmodium*, identify potential new parasite species, and highlight the importance of expanding molecular surveillance in underexplored worldwide ungulate populations.

## 2 Material and methods

### 2.1 Sequences analysed, alignment and filtering

A selection of published cytochrome *b* (*Cyt-b*) sequences of ungulate *Plasmodium* was done to be included in the present study. Sequences were selected to represent the genetic diversity of known *Plasmodium* strains infecting ungulates, with the addition of all “unnoticed” *Plasmodium* sequences of ungulates published by Bitome-Essono et al., 2017 and Mwakasungula et al. (2022). All sequences were aligned with MUSCLE 3.8.31 (Edgar, 2004) and the alignment was cleaned with Gblocks 0.91b (Castresana, 2000) to remove regions with gaps or insertions. In addition, sequences that were too short were excluded from the alignment to ensure sufficient sequence length. After all these steps, the total alignment included 107 sequences and had a length of 740 base pairs (bp), providing a reliable dataset for phylogenetic analyses. The list of all the sequences used is provided in Supplementary Table 1.

### 2.2 Phylogenetic analyses

To infer the genetic relationships among *Plasmodium* parasites infecting ungulates and the other haemosporida, Maximum Likelihood (ML) methods and Bayesian approaches were used for tree construction. For ML, the best-fitting model, based on the Akaike Information Criterion, was GTR (General Time Reversible) + Gamma + I (invariant sites), as determined using ModelTest. The highest-likelihood DNA phylogenetic tree and the corresponding node support values were obtained by using PhyML (freely available on phylogeny.fr platform (Dereeper et al., 2008)), with 1000 bootstrap replicates. To complement the ML analysis, a Bayesian phylogenetic approach was used to determine phylogenetic relationships between sequences, using the software BEAST2 (Bayesian Evolutionary Analysis by Sampling Trees) (Bouckaert et al., 2019). For this inference, a General Time Reversible (GTR) substitution model with gamma-distributed rate variation among sites (+Γ) and a proportion of invariant sites (+I) was also used to ensure comparability between methods. To estimate the posterior probabilities of tree topologies and model parameters, we ran the Markov Chain Monte Carlo (MCMC) analysis for 20 million generations to ensure sufficient sampling of the posterior distribution. Tree sampling was performed every 1000 steps, discarding the first 10 % as burn-in. The convergence of the analysis and the adequacy of posterior distribution sampling were assessed by examining the Effective Sample Size (ESS) values. We verified that all ESS values were greater than 200. This 200 threshold is commonly applied as it indicates that the analysis had run for a sufficient number of iterations to provide reliable estimates (Drummond et al., 2012). The resulting Maximum Clade Credibility (MCC) tree was summarized using TreeAnnotator and visualized and edited using ITOLv7 (Letunic and Bork, 2024).

## 3 Results and discussion

### 3.1 Host diversity of the “unnoticed” ungulate *Plasmodium*

Several new species of ungulates were shown to be infected with *Plasmodium* parasites in the studies of Bitome-Essono et al. (2017) and of Mwakasungula et al. (2022), with new molecular sequences published. In the study performed in Gabon, *Plasmodium* infections were detected and sequenced in three new ungulate host species: *Tragelaphus spekii* (marshbuck), *Syncerus caffe*r (forest buffalo) and *Cephalophus sylvicultor* (yellow-backed duiker) (Bitome-Essono et al., 2017). For the second one, in Tanzania, *Plasmodium* parasites were only detected in the blood of the greater kudu (*Tragelaphus strepsiceros*)(Mwakasungula et al., 2022). Although a previous study had reported infections from one species of the genus *Tragelaphus* (*Tragelaphus spekii*)*,* it concerned only one individual marshubck from Malawi and the parasite was never molecularly analysed (van den Berghe, 1937). Therefore, the two “unnoticed” studies done in Gabon and Tanzania are the first to report and sequence *Plasmodium* infections in the genera *Tragelaphus* and *Syncerus*.

### 3.2 Phylogenetic position and diversity of ungulate *Plasmodium*

**Fig. 1:**
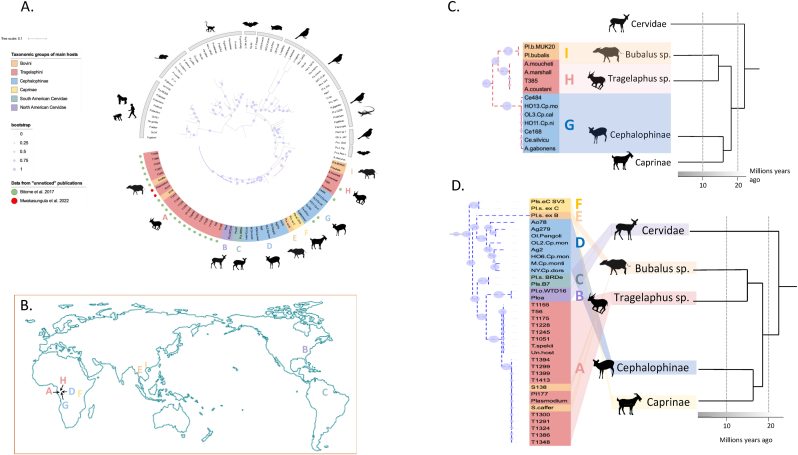
**A-D. Genetic diversity and distribution of ungulate *Plasmodium* discovered so far, including the “unnoticed” parasites. A.** Phylogenetic relationships between haemosporidian parasites based on 740 bp Cytb sequences. Phylogeny was built using Maximum likelihood methods as described in the Materials and Methods section. Red dotted line: Ungulate *Plasmodium* Clade 1. Blue dotted line: Ungulate *Plasmodium* Clade 2. Letters (A–I) below the phylogeny correspond to the different ungulate subclades. Blue dots represent bootstrap values at each node (see legend of the figure). The “unnoticed” sequences of ungulates are indicated by a colored dot (green for Bitome-Essono et al., 2017 and red for Mwakasungula et al., 2022). Representative silhouettes of the main vertebrate hosts are provided for each haemosporidian lineage. **B.** Current known distribution of the different subclades (A–I) of ungulate *Plasmodium* all over the world. **C and D.** Comparison of the phylogenetic relationships between the subclades of Clade 1 and Clade 2, respectively, and the phylogeny of their hosts (simplified from Chen et al., 2019). Colored links connecting the two phylogenies illustrate the host-parasite associations. Concordant speciation events between hosts and parasites suggest a pattern of cospeciation. Host switches to distant relatives are shown by the crossing colored links. An indicative timeline of the estimated divergence between host groups is also provided below each host phylogeny (according to Chen et al., 2019).

Phylogenetic analyses incorporating published sequences, including the “unnoticed” ones, using both Maximum Likelihood and Bayesian approaches, confirmed that ungulate *Plasmodium* parasites form a monophyletic clade. This clade is positioned as a sister lineage to *Polychromophilus* parasites of bats, or to avian or reptilian *Plasmodium* species. This clade is distinct from *Plasmodium* species infecting primates, rodents and bats (Fig. 1A and Supplementary Fig. 1), underlining again the evolutionary divergence of ungulate *Plasmodium* from all other mammal *Plasmodium* species. Results showed that the “ungulate clade” is subdivided into, at least, two well-supported clades. Clade 1, in red in Fig. 1, contains three subclades: one formed by parasites infecting duikers from Gabon, another by parasites found in *Anopheles* mosquitoes and a *Tragelaphus spekii* (marshbuck) from Gabon, and a third lineage with parasites of Asian water buffaloes (*Bubalus bubalis*) (Fig. 1A and B). This clade is well supported with bootstrap values higher than 95 % and a posterior probability higher than 0.99 (Suplementary Fig. 1). The branching order of this clade mirrors the phylogenetic relationships of the different host groups (Fig. 1C), namely a separation between Cephalophinae and Bovinae and then a separation inside the Bovinae between the genera *Tragelaphus* and *Bubalus* (Chen et al., 2019). This pattern could suggest co-divergence of the parasites and their hosts, starting 15 million years ago (Chen et al., 2019). Nevertheless, the highest level of divergence observed between sequences in this clade is lower than levels of divergence observed between *Plasmodium* species that are considered to have diverged more recently (like the *Laverania* parasites infecting humans and African apes). In addition, within the lineage of parasites infecting *Tragelaphus* (subclade H in Fig. 1A), only one parasite has been directly obtained from the host's blood. The remaining parasites in this lineage were identified from *Anopheles* mosquitoes, leaving open the possibility that other host species may also be infected. Consequently, the observed congruence between ungulate and parasite phylogenies could be coincidental. Further research is needed to explore this in more detail.

Clade 2, in blue in Fig. 1, is formed by parasites of Cephalophinae from Gabon, Caprinae from Africa and Asia (with all sequences being genetically identical), Bovinae (*Tragelaphus* and *Syncerus* genera) from Gabon, water buffaloes (from Asia), and Cervidae (white-tail deer of North America and another species from South America: *Ozotoceros bezoarticus*, the Pampas deer) (Fig. 1A and B). This clade is supported by bootstrap values of 79 % and a posterior probability of 1 (Supplementary Fig. 1), but contrary to the other clade (Clade 1), it does not exhibit any evidence of host-parasite co-divergence (Fig. 1D). This suggests a more complex evolutionary history with potential host jumps.

In both clades, the parasites infecting hosts of the genus *Tragelaphus* (with a few also infecting the genus *Syncerus* spp., the African forest buffalo) are distinct from other ungulate parasites discovered and sequenced to date. These parasites form distinct lineages, one of which may correspond to the species previously identified by van den Berghe (1937) in an African marshbuck (*Tragelaphus spekii* formerly known as *Limnotragi spekii*) close to Lake Ihema, in the National Park of Akagera in Rwanda, and named *Plasmodium limnotragi*. In the “unnoticed” parasites, most of them were also found in the blood of marshbucks (*T. spekii*) from Gabon but two specimens were also sequenced from the blood of two kudus (*T*. *strepsiceros*) from the National Park of Ruaha in Tanzania. Phylogenetic analyses of *Plasmodium* sequences isolated from specimens of the genus *Tragelaphus* suggest the presence of at least two distinct *Plasmodium* species infecting these hosts in Central Africa. Regarding the vectors involved in their transmission, these *Plasmodium* species are likely transmitted by *Anopheles* mosquitoes, several of which were found to be infected with parasites closely related to parasites of *Tragelaphus* (Fig. 1A, Clade 1) or were found to be engorged with *Tragelaphus* blood meals (Boundenga et al., 2016; Makanga et al., 2016, 2017).

## 4 Conclusion

The aim of this article was to bring to light information regarding ungulate *Plasmodium* parasites that remained unnoticed by the community of researchers working on it, especially those obtained from *Tragelaphus* hosts. Although these sequences provide new pieces of the puzzle into the diversity and evolution of the ungulate *Plasmodium*, there is still a long path toward a full understanding of what happened in the history of these parasites. The way forward would be to keep exploring the diversity of ungulate parasites in the different continents and extend the variety of hosts screened. This is especially true for Asia and Africa, where other species have been previously described but for which genetic sequences were never obtained. This is the case, for instance, with *P. traguli* that was described in Asian Tragulidae or *Hepatocystis hippopotami* in African hippos (Templeton et al., 2016b). This could be achieved using traditional methods, such as through the screening of blood obtained from bushmeat samples, carcasses or animal captures. Non-invasive methods could also be employed by analysing invertebrate DNA through the use of hematophagous flies (Bitome-Essono et al., 2017; Mwakasungula et al., 2022) or leeches that could be a large source of blood to screen the extant malaria biodiversity in various ecosystems and geographic areas (Drinkwater et al., 2020; Kocher et al., 2017). These innovative methods could significantly enhance our understanding of the complex ecology and evolution of *Plasmodium* parasites in wildlife. The obtention of complete genome sequences of these *Plasmodium* could also allow us to better understand their relationships with other *Plasmodium* as well as the way they adapted to these specific hosts.

## CRediT authorship contribution statement

**Boris Kevin Makanga:** Writing – review & editing, Methodology, Investigation, Formal analysis, Conceptualization. **Larson Boundenga:** Writing – review & editing, Methodology, Investigation, Formal analysis, Conceptualization. **Paul Yannick Bitome-Essono:** Writing – review & editing, Methodology, Investigation, Formal analysis, Data curation. **Céline Arnathau:** Writing – review & editing, Methodology, Investigation, Data curation. **Virginie Rougeron:** Writing – review & editing, Methodology, Investigation, Formal analysis, Conceptualization. **Franck Prugnolle:** Writing – review & editing, Writing – original draft, Methodology, Investigation, Funding acquisition, Formal analysis, Data curation, Conceptualization.

## Conflict of interest statement

The authors declare that there are no conflicts of interest or financial relationships that could have influenced the work reported in this paper.
