# Supplementary material for: Bringing to light unnoticed data on the genetic and host diversity of ungulate Plasmodium
Source: Int J Parasitol Parasites Wildl. 2025 Jun 18;27:101104. doi: 10.1016/j.ijppaw.2025.101104 (PMC12268843; doi:10.1016/j.ijppaw.2025.101104)
Supplement: Multimedia component 1 [file mmc1.docx]

**Supplementary Material.**

**Supplementary Figure 1.** Maximum clade credibility (MCC) tree inferred using BEAST2 for haemosporidian parasites based on 740 bp *Cyt-b* sequence alignment. Phylogeny was built using Bayesian methods as described in the Materials and Methods section. The MCC tree was generated after 20 million MCMC iterations, discarding the first 10% as burn-in. The mean log posterior probability across retained samples was –9591.84 (Standard Deviation = 18.94). All key parameters had ESS > 200, indicating good convergence and sampling. Red dotted line: Ungulate *Plasmodium* Clade 1. Blue dotted line: Ungulate *Plasmodium* Clade 2. Letters (A-I) below the phylogeny correspond to the different ungulate subclades. Blue dots represent posterior probability at each node.

**Supplementary Figure 1.**


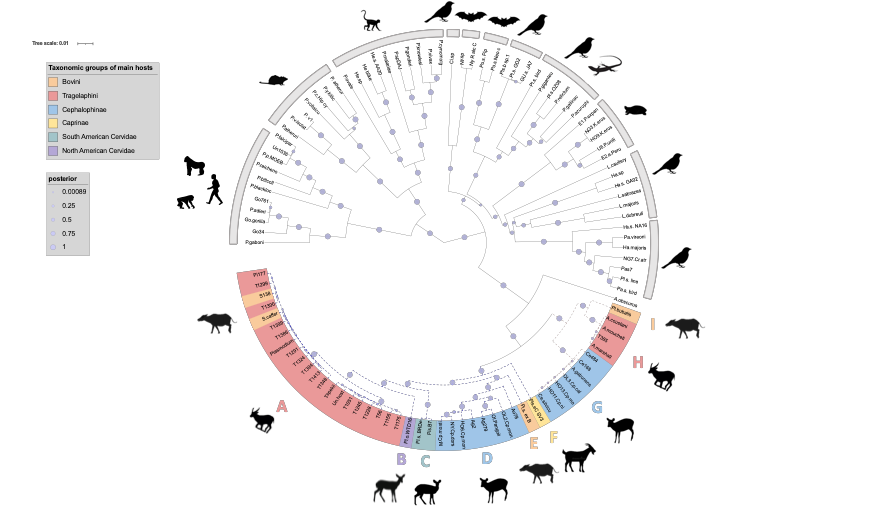


**Supplementary Table 1.** Names of *Cyt-b* sequences used in our study (including the “unnoticed” ones) and their accession numbers. The sequences from Bitome *et al.* 2017 and Mwakasungula *et al.* 2022 are marked with an Asterix (*) or two (**), respectively. Short_names are used in the phylogeny.

**Follow up of Supplementary Table 1.**
